# On network backbone extraction for modeling online collective behavior

**DOI:** 10.1371/journal.pone.0274218

**Published:** 2022-09-15

**Authors:** Carlos Henrique Gomes Ferreira, Fabricio Murai, Ana P. C. Silva, Martino Trevisan, Luca Vassio, Idilio Drago, Marco Mellia, Jussara M. Almeida

**Affiliations:** 1 Department of Computer Science, Universidade Federal de Minas Gerais, Belo Horizonte, Minas Gerais, Brazil; 2 Department of Computing and Systems, Universidade Federal de Ouro Preto, João Monlevade, Minas Gerais, Brazil; 3 Department of Electronics and Telecommunications, Politecnico di Torino, Torino, Italy; 4 Department of Computer Science, Università di Torino, Torino, Italy; 5 Department of Control and Computer Engineering, Politecnico di Torino, Torino, Italy; Unviersity of Burgundy, FRANCE

## Abstract

Collective user behavior in social media applications often drives several important online and offline phenomena linked to the spread of opinions and information. Several studies have focused on the analysis of such phenomena using networks to model user interactions, represented by edges. However, only a fraction of edges contribute to the actual investigation. Even worse, the often large number of non-relevant edges may obfuscate the salient interactions, blurring the underlying structures and user communities that capture the collective behavior patterns driving the target phenomenon. To solve this issue, researchers have proposed several network backbone extraction techniques to obtain a reduced and representative version of the network that better explains the phenomenon of interest. Each technique has its specific assumptions and procedure to extract the backbone. However, the literature lacks a clear methodology to highlight such assumptions, discuss how they affect the choice of a method and offer validation strategies in scenarios where no ground truth exists. In this work, we fill this gap by proposing a principled methodology for comparing and selecting the most appropriate backbone extraction method given a phenomenon of interest. We characterize ten state-of-the-art techniques in terms of their assumptions, requirements, and other aspects that one must consider to apply them in practice. We present four steps to apply, evaluate and select the best method(s) to a given target phenomenon. We validate our approach using two case studies with different requirements: online discussions on Instagram and coordinated behavior in WhatsApp groups. We show that each method can produce very different backbones, underlying that the choice of an adequate method is of utmost importance to reveal valuable knowledge about the particular phenomenon under investigation.

## 1 Introduction

The notion of *collective behavior* has been widely studied in domains such as Sociology and Psychology [1, 2]. A possible definition of collective behavior relates it to “the kinds of activities engaged in sizable but loosely organized groups of people” [3]. Collective behavior emerges in several contexts in both online and physical worlds, and it may drive social, cultural, economic, and political phenomena. For example, groups of users may contribute to disseminating opinions and pieces of information as they produce content in social media applications. These loosely organized people interact with each other driven by common interests and goals or hidden factors (e.g., coordinated actions). Multiple collective behavioral patterns may emerge from such interactions without a predefined social structure that explains them. In turn, those patterns drive and favor an underlying (collective) phenomenon. For example, user discussions around particular topics in a social media application may help disseminate ideas and foster social movements even in the physical world [4–12].

Network or graph modeling is a valuable instrument for studying collective behavior. It provides a set of theoretical tools (algorithms and metrics) that let one identify and characterize an underlying phenomenon of interest [13]. For example, community detection algorithms [14, 15] unveil groups of tightly connected users in a network, letting collective behavior emerge by exploring the topological properties of the network itself [5, 16, 17]. These well-established network metrics and algorithms rely on a graph model where users are vertices connected by edges representing their interactions. Such interactions may involve several individuals simultaneously, e.g., multiple users sharing the same information [8, 10, 18–21] or engaging in discussions around the same posts [22, 23], creating *many-to-many* relationships. To study such systems, edges are often weighted by the number of interactions users have in common [12, 20, 24–26].

In practice, the complexity and diversity of interactions among users pose many challenges to the study of the phenomenon being modeled. Notably, some interactions (i.e., network edges) are of little interest as they emerge sporadically or by chance and do not relate to the phenomenon under investigation [27, 28]. Often in large volume, these edges tend to obfuscate the real underlying structures and user communities representing the collective behavior patterns driving the phenomenon being studied [24, 29]. In other words, the presence of large volumes of irrelevant edges hurts the understanding and interpretability of the given phenomenon. This issue calls for algorithms to remove such edges, a procedure commonly called *network backbone extraction*. The goal of these algorithms is the selection of the edges relevant to a given phenomenon (henceforth *salient edges*) to obtain a reduced and representative version of the network, i.e., the *network backbone*. Given the definition of edge salience is highly subjective, several authors propose different network backbone extraction methods [23, 24, 30–36]. Choosing which of these methods should be applied to a given case study is often a hard task.

A second challenge in the study of collective behavior is the lack of ground truth data, as it is often the case for studies on online social media. Although information about the phenomenon of interest may be present in some scenarios [37, 38], the lack of ground truth prevents the evaluation of the quality of the extracted backbone, making the comparison and choice of methods hard [39–42]. Authors thus resort to the evaluation of topological metrics, such as community modularity, density, clustering coefficient of the extracted backbone, which more clearly defines sub-structures than the original network [20, 37, 43–45].

However, this approach gives no guarantee that the extracted backbone better represents the phenomenon under study. Some recent studies have started exploring regression models to relate topological properties of the network backbone with phenomenon-specific attributes [33, 46, 47]. The intuition is to consider some contextual criteria that relate backbone properties (e.g., the identified communities) to characteristics of the phenomenon under study (e.g., the volume of information shared by a community) [5, 16, 48, 49]. However, these studies do not provide a clear rationale as to why a given extraction method fits the given phenomenon and therefore whether it is adequate to the study. Such reasoning is of utmost importance since different methods have different assumptions and properties, which may constrain their use or introduce unwanted biases to the study.

We here take a step towards filling this gap by presenting a rigorous methodology to select and compare network backbone extraction methods given a phenomenon under study. We introduce a *principled methodology* to compare and select backbone extraction algorithms in the context of online media network analysis. It encompasses two fundamental selection steps: 1) A priori selection of the subset of backbone extraction methods starting from the phenomenon assumptions and the method assumptions; 2) A posteriori evaluation of the resulting backbone’s *structural* and *contextual* quality.

For the first step, we select ten backbone extraction methods, describe their assumptions and requirements, and show that these methods can be grouped into classes to simplify the choice of methods to consider for the particular case study. For the second step, we claim that the traditional topological properties of the extracted backbone graph must be complemented by the evaluation of contextual metrics that assess if the resulting backbone captures the actual correlation related to the phenomenon under study. To exemplify the overall approach, we apply our approach to two specific use cases, each with different characteristics. These cases allow us to show how both steps are fundamental in practice.

Our aim is to offer the researchers a thorough and principled approach when facing the selection of backbone extraction methods. While we present our methodology in the context of online media network analysis, we believe the approach is generic and can be applied in other cases. To foster its application, we make all scripts used to generate our results publicly available, together with two anonymized datasets related to the evaluated case studies in https://github.com/chgferreira/backbone_extraction.

This article is organized as follows. We review the related literature in Section 2 and then formally state the problem we address in Section 3. Next, we describe our proposed methodology in Section 4 and show how it can be applied to two different case studies in Sections 5 and 6. We conclude our paper and discuss potential follow-up studies in Section 7.

## 2 Background

### 2.1 Fundamentals

The modeling of interactions among different users using network/graph representations is widespread in the literature. Yet, as previously shown [24, 28, 50–53], the variety of patterns emerging from such interactions and influencing the phenomenon under study can be quite large, including sequentiality, periodicity, and sporadicity. Moreover, to study a given phenomenon, it is often the case that one needs to look at large sets of interactions over a relatively long period of time. However, not all interactions are equally important for the study. In fact, it is often the case that many such interactions occur only sporadically or purely by chance and therefore have only a weak relation, or no relation at all, to the phenomenon under study.

As previously argued [24, 50, 51, 54, 55] it is unclear the extent to which the aforementioned diversity of interaction patterns affects the study of the phenomenon under consideration. For example, the presence of a large number of random, sporadic and thus weak edges in a network representation may indeed obfuscate those edges more closely related to the phenomenon [10, 12, 20, 56–58], here referred to as *salient edges*. As such, these weak edges represent *noise* to the study. This situation gives rise to the following question: *What makes a particular edge relevant to the study of a given phenomenon?*

As argued by Grady *et al*. [32], the definition of edge salience is based on an ensemble of node-specific perspectives in the network and quantifies the extent to which there is consensus among nodes regarding the importance (or representativeness) of a link. Thus, there are a variety of factors related to the phenomenon under study that define whether an edge is salient or not, making the identification of a salient edge quite challenging. Given a proper definition of edge salience, another challenge is how to implement and evaluate the identification and extraction of these edges from the original network. The set of salient edges is called the network *backbone*. Next, we discuss prior studies that deal with the use and design of backbone extraction algorithms.

### 2.2 Prior studies on network backbone extraction

Starting with applications, multiple works in various fields have shown the importance of backbone extraction methods to deal with random, sporadic, and weak edges that may obfuscate the phenomenon under study. For example, several studies applied early proposed methods to study phenomena in biological networks [59, 60], economic networks [61–63], co-authoring networks [64, 65], human mobility networks [66–68] as well as congressional voting networks [25, 69, 70].

More recently, some studies have highlighted the importance of this task in social media applications. For example, Pacheco *et al.* have proposed a network-based methodology for identifying coordinated actions in social media [20]. This methodology is based on (i) exploring different types of user interactions (e.g., users who used the same hashtag sequence or who retweeted the same tweet sequence) and (ii) applying a threshold-based approach to remove edges whose weights fall below a certain threshold. This simple threshold-based backbone extraction approach has been widely used in the literature [69–74], including studies on online hate communities [12] and communities with online news exposure [10]. However, some studies point to possible misinterpretation of results when using such approach, as they may introduce bias into the analysis [72]. To name a concrete case, the appropriate threshold setting is context-dependent and can therefore be quite complex to be determined. Sometimes it is not even clear whether the same threshold should be applied to all edges [75].

Another example of study is the analysis of information dissemination in publicly accessible groups on WhatsApp, a currently very popular communication platform. The authors of [21, 76] investigated a latent network structure built by connecting users who shared the same content. By applying a probabilistic backbone extraction method called Disparity Filter [77], they revealed the formation of communities of users that cross the boundaries of existing groups, favoring dissemination of information at large [76], including misinformation [21]. Similarly, different backbone extraction methods have been applied to uncover patterns describing the dynamics of online discussions on Instagram, Twitter, Facebook and Reddit [19, 22, 23, 56–58, 78, 79].

Moving to the body of works that focuses on the proposal of new backbone extraction methods, some authors compared methods to alternatives in light of specific phenomena of interest in various domains, such as transportation, finance, and ecology [31, 32, 35, 77]. Some works propose specific rules to the selection of salient edges, e.g., as in [80, 81] where salient edges are identified based on the idea of overlapping communities. A large number of previous studies has proposed and analyzed methods for extracting backbones in *bipartite* networks [37, 38, 82]. Considering a bipartite structure consisting of artifacts and agents, a family of methods, such as the Fixed Sequence Degree Method (FSDM), the Fixed Fill Method (FFM), the Fixed Row Method (FRM), the Fixed Column Method (FCM), and the Stochastic Degree Sequence Model (SDSM), identify salient edges by constraining the degree sequence of either artifacts (FCM), agents (FRM), both (FSDM and SDSM), or neither (FFM).

Most of these studies rely on structural/topological properties to evaluate different backbones extracted from the same network, including node and edge coverage, clustering coefficient, centrality measures, and community quality measures. As such, they offer only a partial view of the quality of the backbones. Contextual (i.e., phenomenon-specific) criteria, capturing the extent to which the extracted backbone represents the phenomenon under study, are not considered. More recently, some studies have proposed and compared backbone extraction methods based on regression-models as a means to capture contextual attributes specific to the phenomenon, relating them to topological properties of the backbone [33, 36, 47].

In this paper, we do not intend to introduce another backbone extraction method, but rather to propose a principled methodology to evaluate existing solutions for a target study. In a similar direction, Dai *et al*. [43] have evaluated six methods for extracting the salient edges from an air transportation network. As most prior studies, the authors considered only topological properties in such evaluation, which seems adequate given the interest in network connectivity (i.e., paths). Similarly, Mukerjee *et al*. [45] investigated the impact of method parameters on network connectivity. They proposed to choose the best method and its parameters based on topological properties, by maximizing the number of edges while maintaining the connectivity of the network. Other studies also evaluated existing methods for specific cases of bipartite networks, but once again considering only topological properties [24, 37, 82].

In contrast, our focus is on collective human behavior in networks emerging in social media applications, which are expected to have more nuanced aspects. Specifically, unlike other networks (such as transportation networks) that are driven by existing and slowly changing rules, our focus is on user behavior, notably on social networks, which is driven by a multitude of both endogenous (e.g., mechanisms employed by the platform) and exogenous factors (interactions on other platforms and in the real world). These aspects deserve to be investigated from both topological and contextual perspectives. We propose a principled methodology to select and evaluate the best method among alternatives for a given target phenomenon, taking into account whether the assumptions and requirements of each method fit adequately the properties of the phenomenon. In the following, we present a brief description of ten backbone extraction methods selected for our study.

### 2.3 Selected backbone extraction methods

In selecting the backbone methods, we consider those that have been proposed for weighted networks and applied to the analysis of collective behavior, often in projected networks [21–23, 31, 33, 35, 56, 64, 76, 77, 79, 83, 84]. We consider methods that have been explored by prior studies, restricting our focus to those applied in social media applications. We include also two alternatives [32, 36] proposed in other contexts to extend the set of to methods.

The selected methods are: naive threshold-based backbone extraction, High Salient Skeleton [32], Random rElationship ClAssifier STrategy (RECAST) [34], Disparity Filter [77], Polya Urn Filter [36], Marginal Likelihood Filter [35], Noise Corrected (NC) [33], Global Statistical Significance (GloSS) Filter [31], Tripartite Backbone Extraction (TriBE) [22], and Stochastic Degree Sequence Model (SDSM) [37].

Here we briefly describe and emphasize the differences among the methods. We provide a short summary of each algorithm in the Appendix A and direct the reader to the original papers where the methods have been proposed for a detailed description.

**Fig 1:**
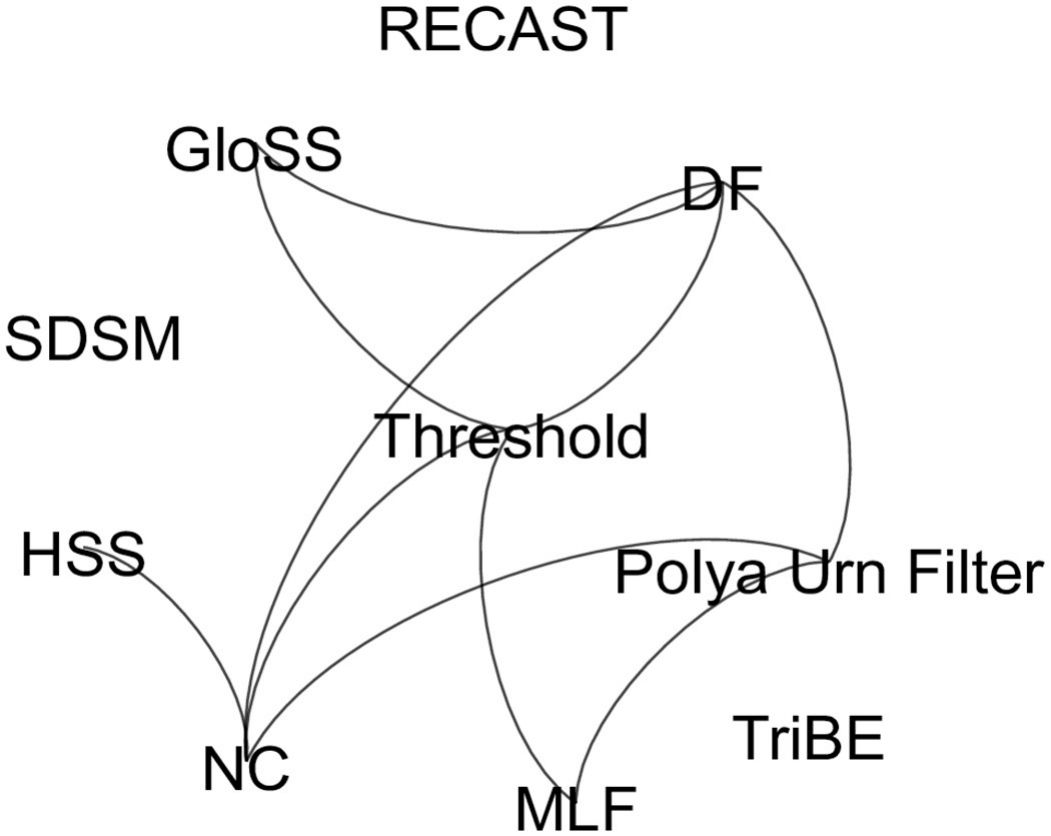
Selected backbone extraction methods: Edges connect methods already compared to each other in prior work. Details of each methods are provided in Table 1.

The aforementioned methods have been analyzed in the context of various phenomena. Yet, no prior study compared all ten methods under the same analysis framework. For illustration purposes, Fig 1 shows which methods have been compared to each other in previous studies mentioned in Section 2.2. Some of them (e.g., RECAST and TriBE) have not been compared to any alternatives. Clearly not all methods are adequate to all studies, which justifies the lack of some comparisons.

## 3 Problem statement

We tackle the challenge of selecting and evaluating network backbone extraction methods available in the literature given a target phenomenon. Inherent to such problem is the use of a potentially *noisy* network to model interactions driving the given phenomenon. By *noisy* we mean a network that may contain a large number of spurious edges that are not relevant for understanding the phenomenon at hand and, even more, may obfuscate the relevant ones (i.e., the salient edges), jeopardizing the understanding of the phenomenon and the validity of conclusions drawn from the study.

### 3.1 Problem statement

Given (i) a particular phenomenon of interest driven by collective behavior, and (ii) a dataset capturing real interactions that represent manifestations of such phenomenon, *how can we evaluate alternative network backbone extraction methods and select the one that, when applied to a network model of the input interactions, is able to accurately reveal key properties associated with the phenomenon of interest?*

One major assumption that guides our effort is that not every network backbone is adequate to study the given phenomenon. Rather, key characteristics of such phenomenon must be matched to the assumptions and requirements of each method. Thus, a characterization of these properties is of utmost importance to drive the analysis. A mismatch between those characteristics, assumptions and requirements may lead to biases and misinterpretations.

Specifically, we are interested in identifying the methods that provide the best agreement between topological properties associated with the connectivity of vertices in the network and the contextual properties associated with factors driving the phenomenon that emerges from those patterns. Since our interest is in collective behavior patterns, the topological properties of interest are mostly associated with *communities* representing tightly connected groups of users who exhibit common behavior. One key challenge we must face is that each backbone extraction method removes edges (and nodes) from the network based on its own definition of edge saliency. Note that nodes that end up isolated after edge removal are also removed from the backbones. Thus, backbones extracted by different methods would reveal different topological structures, with properties that, though possibly strong and clear, may not be relevant (or related) to the phenomenon being studied.

**Fig 2:**
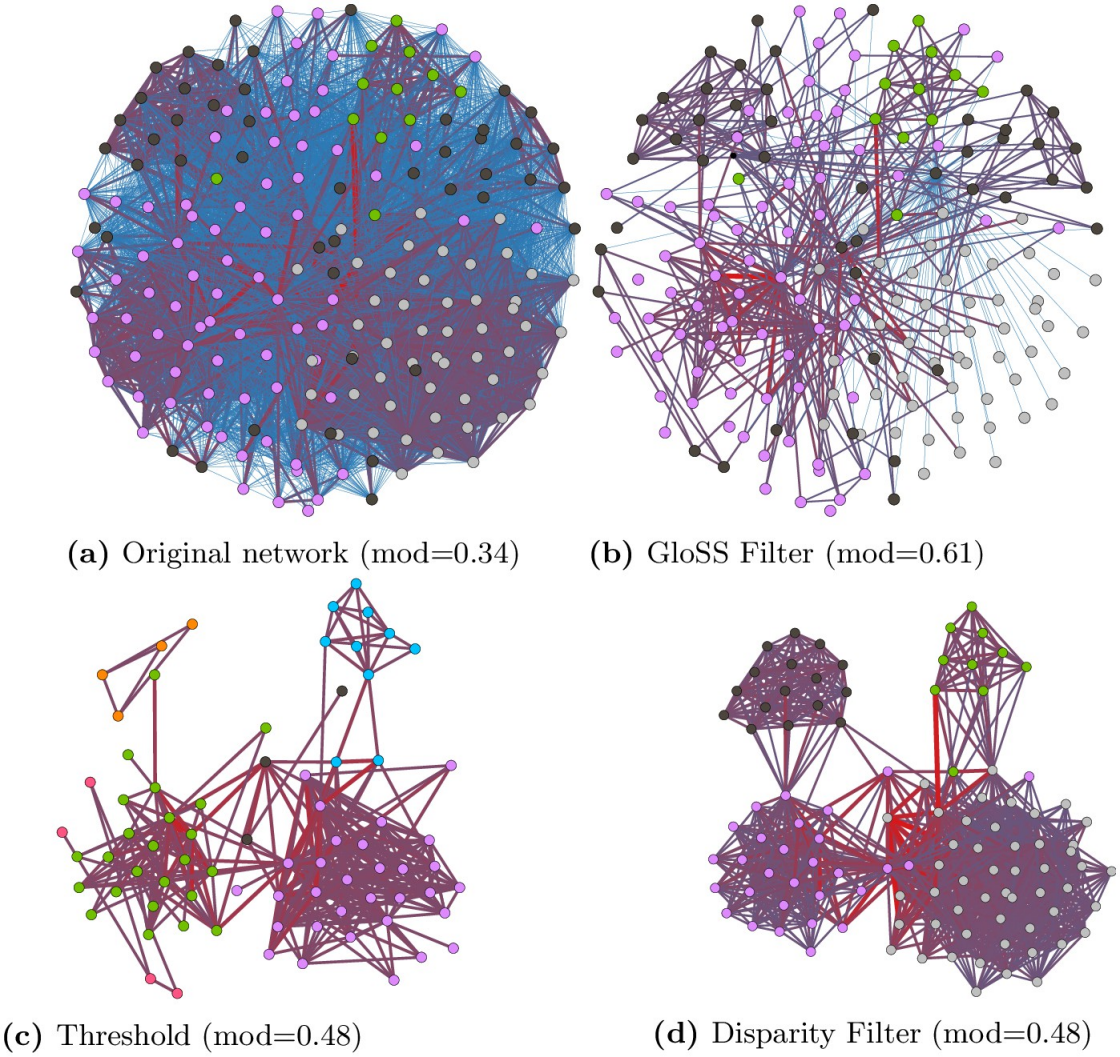
Example network and the backbones extracted from it by three different methods (modularity values presented within parentheses). Edge thickness represents edge weight and nodes’ color possible coordinated users’ communities. Node position is the same as in the original network.

Let us start presenting a simple case to exemplify the complexity of the problem. Consider the network in Fig 2a) built by connecting different users (nodes) who shared the same content on WhatsApp. Edges are weighted by the number of times the users shared the same content. This network, consisting of 190 nodes and 6 760 edges, is a subgraph of the network we analyze in Section 6. Suppose we build this network to investigate evidence of users’ coordination to speed up content spreading on the platform. Fig 2b–2d show three different backbones extracted from the same original network by three different methods, namely the threshold-based method, Gloss Filter and Disparity Filter. As evident, each backbone contains a different subset of the original edges and nodes. The question that arises is: *Which backbone is the best one to study our phenomenon of interest, i.e., coordinated behavior*?

All methods remove a large fraction of the original edges and reveal clear topological structures in terms of communities, which reflected by the modularity [85] which improves w.r.t. the original network (see values in captions of the figures). The Gloss Filter backbone (Fig 2b) is quite different and misses the strong groups of users found by the Threshold and Disparity Filter that form a very tightly connected community. These users shared the same content many times (indicated by the reddest edges), which is a strong evidence of coordination. The spatial layout of the nodes is fixed for all networks. This makes nodes that are members of the same community spread out, which makes it hard to see how interconnected they really are. The threshold-based and Disparity Filter backbones (Fig 2c and 2d) look somehow similar. Both reveal four tightly connected groups of users, but the threshold-based model misses some strong edges among users, resulting in smaller communities. At the end, in this simple case, the Disparity Filter provides the best results.

Note that such conclusions cannot be based solely on topological/structural metrics. Indeed the modularity of the Gloss Filter backbone is the highest, but the extracted communities are not linked with the phenomenon under study. We must thus also consider contextual aspects related to the specific case study.

To generalize and tackle our target problem, in the following we propose a methodology to target the studies of collective behavior emerging from networks. Despite the focus on social media, our proposed methodology is generic enough to be applied to phenomena in other online and offline domains that are also modeled by noisy networks (e.g., co-voting [69, 70] and co-authorship [65] networks).

## 4 Selection and evaluation of network backbone extraction methods

**Fig 3:**
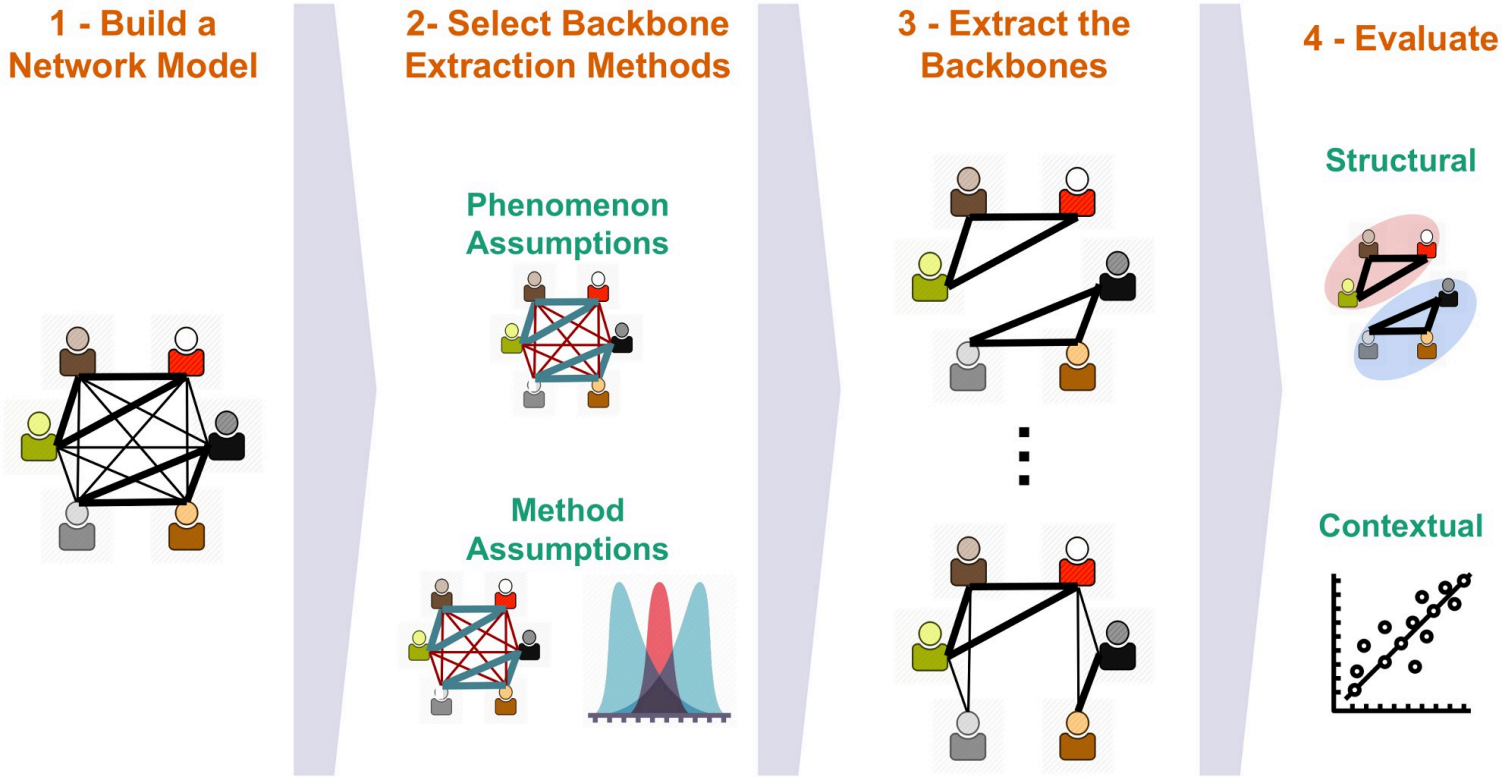
Overall methodology.

The methodology we propose consists of the 4 steps presented in Fig 3. We describe each step in details in the following.

### 4.1 Step 1—Building a network model

We assume the availability of a dataset containing a temporal sequence of user interactions gathered from the target system taking place over a period of interest. In essence, these interactions may occur among multiple users simultaneously, being thus referred to as *many-to-many* interactions. They are observable actions (e.g., comments posted in a social media application) reflecting different user behavior patterns. We are interested in revealing those patterns that are fundamentally related to (and drive) the phenomenon that will be studied.

Such many-to-many interactions can be modeled as a network by building bipartite, a hypergraph or, more broadly, higher order models [53]. Yet, a large number of backbone extraction models work directly on projected networks where edges are added to represent interactions among pairs of users [10, 12, 19, 20, 56–58]. This is the case of Disparity Filter, Noise Corrected, among others [33, 36, 77]. Other methods adopt alternative network models by explicitly representing users as well as phenomenon-specific artifacts through which users interact with each others. As such these methods adopt an n-partite network model. Examples are TriBE that works on a tripartite model representing commenting users, influencers and their posts, and SDSM, which in turn works on a bipartite network representing, for instance, members of congress and voting sessions [22, 26, 70]. However, both methods (as others [26, 37]) end up building a backbone composed of salient user-user edges, thus effectively building a projected backbone. Therefore, we chose to present here the common projected network model. Yet, we note that both Tribe and SDSM operate on the original n-partite network to extract the projected backbone. We thus adopt an undirected and weighted projected network *G* = (*V*, *E*) as the base model for our methodology, such that:

- *V* is the set of users who interacted at least once during the period of interest;
- *E* is the set of undirected and weighted edges connecting pairs of users, such that the weight of edge $e_{i_{1},i_{2}}$ connecting users *i*_1_, *i*_2_ ∈ *V* is *γ*(*i*_1_, *i*_2_) = *f*(*i*_1_, *i*_2_), where *f*(*i*_1_, *i*_2_) is any aggregation function (e.g., count) defined over the set of interactions between *i*_1_ and *i*_2_ and/or any contextual information available associated to them. Examples include the sharing of similar content (e.g., same URLs, same hashtags, or same messages) and/or temporarily synchronized activities [8, 10, 12, 18–20].

Our methodology must give us the means to extract the backbone of the original network where noisy edges are filtered out. Before proceeding, we note that one might be interested on the dynamics of such backbone over different periods of time covered by the input dataset. In that case, one strategy is to break the original data into subsets covering non-overlapping and consecutive time windows (e.g., weeks or months) and build one network model for each window. Given that the phenomenon under study remains the same, it is reasonable to assume that the contextual criteria impacting the selection of the best backbone extraction method would be maintained across network models. Thus, the methodology could be applied to one of such network models to identify the most adequate backbone extraction method. Such method could then be used to extract different backbones (one for each window) allowing an assessment of the temporal evolution of their properties (as done in [21, 22, 25, 55, 69, 70, 76, 86]).

### 4.2 Step 2—Selecting candidate backbone extraction methods

In principle, any backbone extraction method could be applied to a given network model, and the backbones extracted by different methods may be quite different (as illustrated in Fig 2). Some backbones may miss a few important edges while still offering important insights, whereas others may be composed mostly of edges of little relevance to the study. Detecting the latter is not always easy, especially for large-size networks. Thus, we argue that a careful and principled selection of candidate methods must be performed *before* evaluating the extracted backbones to avoid misinterpretations and facilitate evaluation. To that end, our goal in this step is to shortlist backbone extraction methods that are adequate to study the given phenomenon. By adequate we mean that their assumptions and requirements are in alignment with key characteristics of the phenomenon, at the cost of generating completely unrelated backbones otherwise.

In the following we offer a characterization of ten alternative methods (cfr. Section 2.3) and discuss issues one must consider to study a target phenomenon. The discussion below reflects our analyses of the methods’ applicability to different scenarios. To guide this discussion, in Table 1 we present a summary with some key properties of each method. We categorize the methods along with four aspects that are important to assist one in determining the suitable methods for a given case study.

**Table 1:** Our characterization of selected backbone extraction methods.

Method	Edge weight Domain	Edge Salience Criteria		Parameters
		Local vs. Global	Structural vs. Statistical	
Threshold-based Backbone Extraction	Positive/negative continuous or discrete	Global	Structural	Threshold (Edge Weight) or % Edges
High Salient Skeleton (HSS) [32]	Positive continuous or discrete	Global	Structural	Threshold (% Edges or HSS Score)
RECAST [34]	Positive discrete	Global	Statistical Reference model: Two global distributions for all edges from random graphs with the same topology as the original network	*alpha* (significance level)
Disparity Filter (DF) [77]	Positive discrete	Local	Statistical Reference model: Uniform distribution of edge weight per node	*alpha* (significance level)
Polya Urn Filter [36]	Positive discrete	Local	Statistical Reference model: Beta-Binomial distribution of edge weight per edge	*alpha* (significance level) and *a* (Reinforcement Learning)
Marginal Likelihood Filter (MLF) [35]	Positive discrete	Local	Statistical Reference model: Binomial distribution of edge weight per edge	*alpha* (significance level)
Noise Corrected (NC) [33]	Positive discrete	Local	Statistical Reference model: Binomial distribution or a Hypergeometric distribution (obtained by a Bayesian Framework) of edge weight per edge	*alpha* (significance level)
Global Statistical Significance (GloSS) [31]	Positive continuous or discrete	Local	Statistical Reference model: A single null model considering both the edges between nodes and the weight distributions of the original network. Each edge is evaluated under its end nodes’ properties using a Bayesian Approach	*alpha* (significance level)
Tripartite Backbone Extraction (TriBE) [22, 23]	Positive discrete	Local	Statistical Reference model: Poisson-Binomial distribution of edge weight per edge	*alpha* (significance level)
Stochastic Degree Sequence Model (SDSM) [37]	Positive discrete	Local	Statistical Reference model: Poisson-Binomial distribution of edge weight per edge considering the bipartite degree sequence	*alpha* (significance level)

Before we look at the edge salience criteria, the first aspect that should be considered when deciding which backbone method to apply is the nature of the edge weights (2^*nd*^ column of Table 1). This preliminary aspect is a fundamental step in our proposed methodology to discard methods that are not applicable to the problem. Most of the methods considered here are limited to discrete weight values (Noise Corrected, Disparity Filter, TriBE, RECAST and SDSM), while others work with continuous values too (HSS, Threshold and GloSS). Similarly, another factor to be considered is whether the method expects only positive weights—e.g., only the Threshold-based approach can work with negative weights among the ten evaluated methods.

#### 4.2.1 Global vs. Local methods

The second aspect is inherently related to how the method determines whether an edge is salient or not (3^*nd*^ column of Table 1). While some methods apply a single criterion to all edges, others may use different criteria for different edges. Thus, we propose to classify each method as either *local* or *global*. The former refers to methods that determine the salience of each edge based on local information associated with the neighborhood of the edge, thus capturing aspects that are specific to the edge (and adjacent nodes) being analyzed. Global methods, instead, use the entire graph or a single global property for all edges in the graph. As such, the same (global) criterion is applied to all edges. As shown in Table 1, the simple threshold-based backbone extraction, HSS and RECAST are global methods. All other seven methods are local. It is important to note that while GloSS uses a single reference model, the selection of salient edges is based on local information about the degree and strength of adjacent nodes [31].

The choice between a local or a global method should take into account whether the phenomenon exhibits an inherent heterogeneity or possible biases across different edges that are relevant to the understanding of the phenomenon. For example, it is well-known that several attributes related to user behavior in social media applications (e.g., content popularity, content sharing etc.) are very heterogeneous, resulting in heavy-tailed distributions [87, 88]. Such distributions naturally lead to network models with edge weights, node strengths and other properties that are widely distributed, often over different scales [13, 89, 90]. If the phenomenon under investigation is inherently related to a single (dominant) scale (e.g., revealing the most frequent interactions) or to properties that go beyond single edges and their adjacent nodes (e.g., revealing users who can easily reach all others in the network), then a global method should be adequate.

Otherwise, if the phenomenon occurs at all scales defined by the heterogeneous structure of the network, a local method is probably more adequate. By exploring local information to define the salience of an edge, such methods might be able to retain edges that are representative of multiple scales, thus being relevant to the phenomenon. One such example is the study of online discussions in social media. Participation in such discussions is naturally highly heterogeneous reflecting the differences in user behavior. Yet, to get a clear picture of what is being discussed, one must capture the contributions of users with different levels of activity. Applying a global method may bias the extracted backbone to the interactions among the most active users or the most popular content, which would offer only a partial view of the discussions. A local method, instead, would be able to retain interactions among users with different levels of activity, thus offering a more complete and accurate representation of the interactions driving the phenomenon. We further elaborate on this particular study in Section 5.

#### 4.2.2 Structural vs. statistical methods

A third aspect to be considered is whether edge salience is based on structural properties or on a statistical reference model (4^*rd*^ column of Table 1). The former relates to methods that determine whether an edge is salient based solely on topological attributes of the network (e.g., edge weights, neighborhood overlap, paths etc.), thus relying only on the empirical distributions of these attributes. These distributions are often evaluated via thresholds. As shown in Table 1, both the threshold-based and HSS methods fall into this category. For the former, salient edges are those whose weights are above (or below) a given threshold. For the latter, the number of shortest path trees that use the edge is used as attribute. Structural methods are more adequate if the phenomenon is inherently related to the network topology or connectivity, as represented by the used attribute. Examples include revealing the interactions among users/nodes with the largest number of neighbors in common (highly neighborhood overlap) [69, 70], or revealing users who are sources of information with greater reach in the network [91].

In contrast, other phenomena may be studied in more details by examining statistical deviations from an expected reference behavior. In such cases, one should consider methods that build statistical reference models for edge weights. These methods consider as salient the edges whose weights deviate significantly (according to a given *alpha*) from the reference model. The idea is that such reference model reflects the random network structure that would emerge if the phenomenon would not be taking place. As such, it is built based on network properties (e.g., distribution of node degrees, node strengths, or edge weights) often under the assumption of independent user behavior. By looking at edges that statistically deviate from the reference, these methods avoid uninteresting (common) behaviors, thus focusing on the edges that have greater chance of reflecting uncommon interactions that drive the phenomenon under investigation.

Different methods employ different reference models, thus directly impacting the definition of salience. To select a method, one should consider whether the employed reference model reflects a baseline for analysis. Consider, for instance, the study of coordination among users to spread information where interaction occurs when two users share the same content. A strategy to model this phenomenon is to consider that users should have similar sharing patterns with their neighbors in the network if no coordination is taking place. This behavior leads (as the reference model) to a uniform distribution of edge weights for all edges incident to the same node. Edges with weights that significantly deviate from such reference offer potential evidence of coordination and, thus, should be retained as part of the backbone. We further elaborate on this study in Section 6.

#### 4.2.3 Parameters to filter

The fourth aspect relates to parameters employed by each method (5^*th*^ column). As shown in Table 1, all structural methods rely on a threshold parameter to determine salient edges. As mentioned in Section 2, the use of such approach may lead to biases in the analyses. To avoid such problems, a threshold can be set contextually, i.e., based on an expected value for an edge according to the phenomenon. Since setting the threshold based on a contextual decision may be quite complex, prior work has proposed to consider a percentile of the empirical distribution of weights, analyzing the impact of this value on topological properties, e.g., density and community quality [24, 69, 74].

Conversely, all statistical methods make use of a parameter *alpha* for statistical testing to identify salient edges. Typically, the literature uses classical values (i.e., 0.1, 0.05, 0.01 or 0.005 or 0.001) [31, 32, 36]. However, some studies have argued that such classical values do not always yield the best topological structure of the network [24, 45]. Moreover, methods yield different reference models, some of which provide more tighter estimates than others. Thus, we propose here to test a range within these values to examine the effects on both topological and contextual properties, as we will explain later, and to choose values that represent a good compromise between the two metrics for each method. In addition to the parameter *alpha*, the Polya Urn filter also requires a parameter *a* that governs the process of reinforcement of existing interactions [36]. The higher the value of *a*, the larger the weight of an edge between two nodes must be, compared to the weights of the other edges adjacent to those nodes, for the edge to be considered salient.

#### 4.2.4 Additional considerations

Having discussed the aspects that must be considered when selecting backbone extraction methods, we complete this step with some general considerations and insights about specific methods that may also help guide the selection. First, we note that some of the local statistical methods, notably TriBE, SDSM, MLF and NC, use binomial or Poisson binomial distribution as reference model for edge weights. These statistical distributions assume—by design [92]—that each unit of edge weight is assigned to a pair of nodes under the assumption of *independence*. Deviations from this assumption are considered relevant evidence of salience in the context of social media applications, as they suggest that the weights are generated by hidden effects, e.g., when users are attracted to certain content and therefore interact around them [22, 47]. The Polya-Urn filter, on the other hand, assumes the beta-binomial distribution which breaks with the assumption of independence since each assignment is not independent of the others and changes from trial to trial (see section 2).

Moreover, recall that social media applications are characterized by a great degree of heterogeneity in user activity and content popularity. TriBE, being designed for this context, captures such heterogeneity directly, by using these factors to build the reference model. In contrast, the SDSM method captures these aspects through the corresponding degree sequences in the bipartite graph. By setting these properties, the degree at the top (artifacts) of the bipartite network represents content popularity while the degree sequence at the bottom (agents) represents users activity level. MLF and NC capture that indirectly, by considering node degrees and node strengths to build the reference models. Intuitively, these node attributes are closely related to user activity and content popularity. On one hand, as very active users tend to interact more with others, the degrees and strengths of the corresponding nodes in the network tend to be larger. Similarly, more popular content tends to attract more users, thus contributing to increasing the strengths and degrees of the corresponding nodes. GloSS filter also uses the same attributes to determine whether an edge is salient, though using a somewhat different approach. Therefore, all these five methods share similarities in terms of the definition of edge salience, producing backbones that include edges with great variety of weights.

In contrast, the other evaluated methods explore network heterogeneity in the sense that edges with larger weights, either from a local (Polya Urn and DF) or a global (RECAST, HSS and threshold-based) perspective, are more likely to be salient. Both Polya Urn and DF build different reference models to seek edges that stand out (from a local point of view) by their weights considering a subset of nodes/edges. HSS and the threshold-based method, instead, take a global perspective (the structure of the whole network or a target threshold) as reference to identify salient edges. RECAST, in turn, characterizes edges into four classes, allowing different definitions of edge salience (see Section 2). Yet, by exploring such classes, namely *Friends* and *Bridges*, one may produce backbones that also favor edges with heavier weights. In short, in some cases structural and statistical methods can capture similar behaviors (e.g., Threshold vs. Disparity Filter and Polya Urn), but in other cases they capture completely different behaviors (e.g., Threshold vs. GloSS Filter). Thus, the choice of methods depends primarily on the domain and the context. Considering that the network model we build encodes user interactions, such methods favor keeping edges in the backbone based on repetitive and consistent patterns of interactions.

### 4.3 Steps 3 and 4—Backbone extraction and evaluation

Having identified a set of backbone extraction methods that could be employed in a particular study, step 3 consists of applying the selected methods to the original network to extract the corresponding backbone. Specifically, each candidate method *c* in a set of methods *C* identified in step 2 is applied to extract a backbone $B^{c}=\left(V_{b}^{c},E_{b}^{c}\right)$, such that $E_{b}^{c}\subseteq E$ consists of only edges considered salient by *c* and $V_{b}^{c}\subseteq V$ is the set of nodes with at least one edge in the backbone extracted by method *c*. In other words, after backbone extraction, all isolated nodes are disregarded. Step 4 consists of evaluating the quality of the produced backbones. In case multiple methods were selected in step 2, the best alternative should be chosen according to a trade-off between the metrics discussed next. The backbone produced by the best method would then be used to carry out the study.

Building on prior work [33, 36, 47, 64, 85, 93], we consider metrics of backbone quality in two categories: topological, which are closely related to network and community structure, and contextual, which refers to phenomenon-specific attributes.

#### 4.3.1 Topological metrics

The topology-related metrics aim at quantifying the extent to which the network structure emerging from the backbone provides a clear view of how users are organized. Metrics such as node degree, density, clustering coefficient, number of connected components, modularity (see discussion below) characterize the structural properties of interactions considered as salient by the backbone extraction process. For the sake of brevity, we refrain from formally presenting all such metrics here and refer the reader to [94] for formal definitions.

Recall that our main focus is on phenomena related to collective user behavior. Examples in the social media domain include efforts to promote particular ideas, brands, or ideologies. The graph concept that can be directly applied to this notion of collective behavior is *community*. Thus, the emergence of clearly defined (i.e., strongly structured) communities in the backbone offer potential evidence of groups of users actively engaging in common behavior. Identifying such communities is an important step to uncover relevant knowledge about the phenomenon [21–23, 25, 64, 70, 74, 76].

The community detection literature is quite extensive, with approaches focusing on specific concepts of communities defined over different network models [48, 95, 96]. However, in general terms, the definition of a community naturally implies groups of users who are more *similar* with respect to common interactions and other behavioral patterns. Therefore, users in a given community are more strongly connected to each other than to the rest of the network. We chose to apply the Louvain algorithm [85, 97] to identify communities in the backbones, as it is one of the most used algorithms for community detection. Yet, this component of our methodology could be changed to employ alternative methods such those described in [96]. The goal of the Louvain algorithm is to maximize the *modularity* of the communities.

Intuitively, the modularity captures how much densely connected the nodes within a community are, compared to how connected they would be in a random network with the same degree sequence. Modularity is defined in the range of -0.5 to +1, and modularity scores of 0.3 or higher are considered strong evidence of well-shaped communities. The Louvain method is a heuristic that operates by finding first small communities optimizing modularity locally on all nodes. Then, each small community is merged into one meta-node and the first step is repeated. The final number of communities is the result of an optimization procedure. We refer the reader to [97] for a detailed description of the Louvain algorithm.

#### 4.3.2 Contextual metrics

In addition to topological metrics, the quality of a backbone should be assessed with respect to how well it represents properties of the phenomenon. For example, by focusing on communities and, in particular, by exploring the contextual properties associated with them—i.e., characteristics of the communities that are not explicitly captured by the network topology, but are intrinsically related to the phenomenon—we may uncover properties that can help explain the emergence of different collective behavior patterns. In this way, we can gain insights into factors driving the phenomenon [5, 16, 48, 49].

Unlike topological attributes, contextual criteria of backbone quality require additional information about the phenomenon. For example, in the case of social media applications, contextual information can be obtained through metadata that is usually collected when studying these applications. We thus also propose to assess how well the backbone captures phenomenon-specific properties by means of regression models. Specifically, we build upon prior work [33, 46, 47], where contextual (phenomenon-specific) properties are used as explanatory variables to build linear regression models with edge weights as the response (dependent) variable.

Although only linear regression models have been used in these previous studies, nonlinear models could also be considered. They are particularly appropriate when the chosen covariates are known or expected to have a nonlinear relationship with the edge weights. One could consider, for example, the task of jointly predicting the edge weights using Exponential Random Graph Models (ERGM) or even Graph Neural Networks, despite some limitations of the ERGMs when it comes to estimating parameters from sampled graphs (see [98] for a deep discussion).

Driven by the case studies presented in Sections 5 and 6, we restrict ourselves to linear models to estimate the edge weights in the backbone (or in the entire network). Since our goal is to compare the ability of backbone extraction models in removing spurious edges, we argue that measuring the accuracy when estimating individual edge weights is a good proxy for performance in this task. Specifically, we consider the following regression model:

$$\begin{array}{c} \gamma \left(i_{1},i_{2}\right)=\beta_{0}+\beta_{1}X_{1}+\beta_{2}X_{2}+\ldots +\beta_{n}X_{n}+\epsilon , \end{array}$$ (1)

where *γ*_(*i*_1_, *i*_2_)_ is the weight of $e_{i_{1},i_{2}}$, *X*_1_…*X*_*n*_ is a set of covariates related to the phenomenon, *β*_0_…*β*_*n*_ are the model coefficients and *ϵ* is an error factor.

The quality of the model fitted to the data captures how well the covariates (contextual properties) can be used to explain the edge weights (topological property). The better the fitting of the regression model, the more representative the considered edges (and corresponding weights) are of the underlying network structure driving the target phenomenon. In particular, we expect that the fitting of the regression model produced for the edges in a backbone (i.e., only edges in *E*_*b*_) to be better than the fitting of the model produced using the entire (noisy) network (i.e., all edges in *E*). Similarly, we can compare the quality of different backbones by comparing the fitting of the models produced for them.

Although this approach has been used in previous studies [33, 46, 47], we point out some limitations. First, prior work only considered as a quality measure the coefficient of determination *R*^2^, or its relative improvement for the backbone over the original network. However, *R*^2^ values may be misleading as they do not account for error estimates [99]. Therefore, we propose to assess the quality of the fitting by using both *R*^2^ and the root mean square error (RMSE), which is the square root of the mean squared difference between estimated and observed values [100]. Smaller RMSE values suggest better (i.e., more accurate) fittings of the model. To compare RMSE values for different networks/backbones, we use a normalized version of RMSE, where edge weights are normalized by the average value. We recommend the following references for more details [99–101].

Another issue is that backbones extracted by different methods may be quite different in terms of both the number of salient edges and the ranges of weight values, as the methods may favor very different edges during selection of the salient ones. On the one hand, one would like to assess the quality of each backbone using all (or most of) its edges. On the other hand, it may be interesting to compare different methods over the same set of (salient) edges. As a trade-off between these two scenarios, we propose to split the data into a training and a test set, whereas the latter consists of a smaller set of edges common to all backbones. We then evaluate the backbone quality in both sets.

Specifically, we first identify the largest common set of edges present in all extracted backbones $E_{b}^{\cap }={⋂}_{c\in C}E_{b}^{c}$, where *C* is the set of alternative backbone extraction methods to be evaluated. We then propose to randomly select a sample *T* of $E_{b}^{\cap }$ as *test edges*. We choose to select 20% of $E_{b}^{\cap }$ as test edges, but other sample sizes could be adopted [102]. Next, for each method *c* ∈ *C*, we build the regression model using all edges in $E_{b}^{c}\backslash T$, that is, all edges in the extracted backbone except those in the testing set are used as *training edges*. We do the same for the entire network, using set *E* − *T* as training edges.

We first evaluate the quality of each regression model using both *R*^2^ and NRMSE over the *training edges* to assess how well the model fits the training data. Note that the training data captures the majority of the edges in the original backbones. As such, by analyzing the model fitting to this data we are able to assess the extent to which each backbone is indeed capturing relevant information for the phenomenon under study.

We then assess the quality of each model in the common set of test edges *T*. That is, we use the trained models to estimate the weights of edges in *T*, and evaluate the quality of the fitting using NRMSE. We only use the NRMSE because it is better suited for checking how far the points of the common test set are from the regression line [100]. In a sense, *T* captures the consensus in terms of edge saliency among all methods. As such, we note that similar NRMSE values in the training and test sets for a given method suggests that this consensus is representative of the entire backbone extracted by the method. In contrast, larger NRMSE values in the test set suggests that the backbone extracted by the method deviates significantly from the other backbones (that is, the test set is not representative of the training data).

Note that we chose to use a sample of $E_{b}^{\cap }$ as test edges, instead of the complete set, to avoid favoring particular methods. For example, backbones with larger relative intersections with $E_{b}^{\cap }$ (i.e., the smaller backbones) could be favored in the quality assessment as edges in $E_{b}^{\cap }$ are more representative of the training data. Indeed, we did observe this effect in a preliminary set of experiments when the complete set $E_{b}^{\cap }$ was used as test edges in our case studies. This effect has been reduced as we adopt the strategy of using a sample of $E_{b}^{\cap }$ as test edges instead. This strategy has also the side effect of leaving more edges to build the model, which may lead to more accurate models.

#### 4.3.3 Deciding on the best parameters and methods

One question remains: *How to select the best parameters for each method, which indeed may change the obtained backbones and, as such, the respective topological and contextual metrics for the method?* The final decision on which methods to take depends on these choices. The selection of the best parameters is a multicriteria optimization, in particular for the statistical methods. It should take into account the backbone quality in terms of both topological and contextual perspectives (i.e., modularity, *R*^2^ and NRMSE) as well as node and edge coverage. As argued above, for the sake of a fair comparison among methods, we create a common set of edges across the extracted backbones out of which we build a common (sub)set of test edges. Similar regression values in the training and test sets for a given method suggests that the common set is representative of the entire backbone extracted by the method, while divergent results indicate that the backbone extracted by the method differs significantly from the others. Thus, an important constraint on the selection of parameter values is the size of the common set of backbone edges for all methods. Given that the methods differ greatly in terms of how aggressively they remove edges/nodes as well as in the quality of the extracted backbones, we must look for a compromise.

Our strategy is to start with the most aggressive method and choose a value for *alpha* that delivers the best trade-off between modularity and regression results while still leaving a large subset of edges. We then proceeded with the following methods in decreasing order of aggressiveness, potentially reviewing our previous choices in case the common subset of edges becomes small.

Finally, although here we focus on the quantitative assessment of backbone quality, one could also resort to visualization to identify possible differences between backbones extracted by different methods, especially on the denser components of each backbone. This is important because both the topological and the contextual perspectives are subject to approximations and should therefore be considered complementary. In addition, one should be aware that backbone analysis from a contextual perspective, as opposed to a topological perspective, inevitably includes subjective factors (e.g., the selection of predictor variables).

## 5 Case study 1: *Online discussions* on Instagram

### 5.1 Characterization of the phenomenon

Our first case study focuses on *online discussions on Instagram*. In this social media application, top public profiles (called “influencers”) create *posts* that attract the interest of users, who may *like* or leave *comments* associated with them. Specifically, Instagram users engage in *online discussions* by commenting on posts [22, 23, 103–107]. The study of such discussions can offer important insights into how information is disseminated on the platform and how the online debate impacts our society [108–113].

We rely on a sample of the dataset gathered and analyzed in our earlier work [22, 23]. It is composed of public content published by political influencers in Brazil in the week surrounding the first round of Brazil’s 2018 general elections (e.g., from September 30th to October 6th). We gather posts by the eight candidate runners: @jairmessiasbolsonaro, @fernandohaddadoficial, @lulaoficial, @cirogomes, @_marinasilva_, @guilhermeboulos.oficial, @cabodaciolo, @ad.alvarodias. We use all posts created by these profiles during the election week, as well as the comments they received from other Instagram users. We choose not to include users who commented on a single post, as these clearly reflect sporadic behavior. In total, we analyze 41099 users who made 376779 comments on 540 posts.

### 5.2 Step 1—Building the network model

We model the interactions among users commenting on the Instagram posts using the network model proposed in [22, 23]. This network model is defined as a weighted undirected graph *G*_*Instagram*_ = (*V*, *E*), where:

- *V* is the set nodes representing the users who commented on posts; and
- *E* is the set of edges, where each edge (*i*_1_, *i*_2_) ∈ *E* connects nodes *i*_1_ and *i*_2_, representing two users who commented on the same post. The edge weight *γ*(*i*_1_, *i*_2_) is the number of posts that received comments by the same two users.

Many interactions represented by edges in this network model may not reflect *actual discussions*. For example, a very popular post naturally attracts many users, who comment on them often in an independent manner, without actually engaging in a discussion about the topic. Moreover, some users are more active than others. Such cases lead to the emergence of a number of edges simply *by chance*. As these edges do not reflect discussions among the users/commenters, they represent *noise* to the study of the target phenomenon.

### 5.3 Step 2—Selection of candidate backbone extraction methods

The understanding on how the inherent properties of the phenomenon impact the network model helps us to focus on backbone extraction methods that do take such properties into consideration to identify salient edges. In this context, salient edges are those with greater evidence of reflecting online discussions. Hence, we are looking for methods that consider the effects of *user activity level* and *post popularity* on the emergence of irrelevant edges. As such, methods that are based on the assumption that edge salience is necessarily related to edge weight (e.g., methods that assume that edges with larger weights are more likely to be salient), either from a local or a global perspective, are not adequate. These methods tend to retain in the backbone only edges representing repetitive patterns of the most active users, disregarding interactions reflecting discussions carried out by less active (though still important) users.

Given these considerations, we select the following set of candidate methods for further evaluation: *C* = {MLF, NC, Gloss Filter, TriBE, SDSM}. These methods are fundamentally local and statistical, and factor both *user activity level* and *content popularity*. TriBE and SDSM explicitly build a reference model based on both characteristics. MLF, NC and GloSS Filter evaluate the salience of an edge taking these factors into account indirectly, by exploring structural aspects of the network, notably node strength and degree, which are affected by user activity level and content popularity (cfr. Section 4.2.4).

Notice that although we argue that the SDSM method is adequate to the study of online discussions [37], it has some theoretical advantages and disadvantages with respect to the other methods. First, while SDSM explicitly controls the degree sequence of both artifacts and agents, it does not consider that some artifacts can only have links to certain subsets of agents. In the context under study, this implies the assumption that all 41 k users (i.e., agents) can comment on all 540 posts (i.e., artifacts). Even though this is possible in theory, it does not happen in reality, as users select specific posts and influencers to comment on [22, 23]. Thus, the expected value of an edge between two users is underestimated, making it more likely that edges are considered salient. In contrast, the other considered methods, which work on a projected network (i.e., [23, 31, 33, 35]), allow for disjoint subsets of commenters per post as only the actual edges (which capture user preferences) are represented in their reference models. In particular, TriBE [22, 23] was designed to capture such inherent heterogeneity of user behavior and preferences. It does so by considering user engagement towards posts of specific influencers, i.e., a disjoint relationship between subsets of artifacts (posts) and subsets of agents (users). In other words, a tripartite structure is considered, which is able to compute different distributions for a subset of users and posts and thus more accurately determine the expected value of an edge.

### 5.4 Step 3—Backbone extraction

We apply each candidate backbone extraction method in *C* to the network model built in step 1. Recall that all the selected methods require *alpha* as parameter, and we test a range of possible values. Table 2 summarizes the topological properties of the original network and the backbones extracted using the considered methods. The reported backbone results were obtained with the following *alpha* parameters: TriBE = 0.05, SDSM = 0.001, GloSS = 0.10, MLF = 0.001, and Noise Corrected = 0.00001. Results for other values of *alpha* are reasonably consistent—see Table 6 in Appendix B. Recall that we chose *alpha* that offered the best tradeoff between modularity and regression results while still producing a large subset of common edges across methods.

**Table 2:** Online discussions on Instagram: Topological metrics of the network and backbones extracted by each candidate method. Columns 2-3 contain total numbers for the original network and also the corresponding percentages for backbones.

Network Model	Nodes	Edges	Avg. Deg.	Density	Avg. Clust.	# C.C.	# Comm.	Mod.
Original network	41 099	1 193 201	9 236	0.2248	0.68	1	4	0.25
TriBE’s backbone	40 816 (99.3%)	4 152 501 (2.1%)	203	0.0050	0.30	1	9	0.56
SDSM’s backbone	31 811 (77.4%)	2 794 969 (1.4%)	175	0.0055	0.33	2	10	0.46
GloSS’s backbone	28 065 (68.2%)	4 891 339 (2.5%)	348	0.0124	0.54	1	7	0.32
NC’s backbone	28 459 (69.2%)	4 285 139 (2.2%)	301	0.0106	0.31	1	7	0.61
MLF’s backbone	20 461 (49.7%)	2 195 999 (1.1%)	214	0.0105	0.33	1	7	0.62

Columns 2-7 in the table show the results for topological metrics: nodes and edges (total numbers for the original network and also corresponding percentages remaining in the extracted backbones), average degree (Avg. Deg.), density, average clustering coefficient (Avg. Clust.) and the number of connected components (# C.C.). Due to the high execution time, we use 20% of the nodes in each network to estimate the clustering coefficient. Overall the backbones are sparser than the original graph, since a large fraction of the nodes and edges have been removed. Moreover, the average clustering coefficient shows a moderate number of connected triangles in the network for all methods. Interestingly, four out of the five resulting backbones have only one connected component, as is also the case for the original graph, suggesting the presence of key users promoting online discussions across different Instagram profiles and connecting salient edges into a single component.

The three rightmost columns of Table 2 show results of community-related metrics. We consider only communities with more than 100 users. The number of communities (# Comm.) is larger than in the original network. This is expected since backbones are sparser than the original graph. Finally, the rightmost column of Table 2 shows the modularity results as a measure of community quality. All backbones clearly have more strongly connected communities (i.e., higher modularity) than the original network, although we still need to assess the representativeness of these backbones using contextual information.

**Fig 4:**
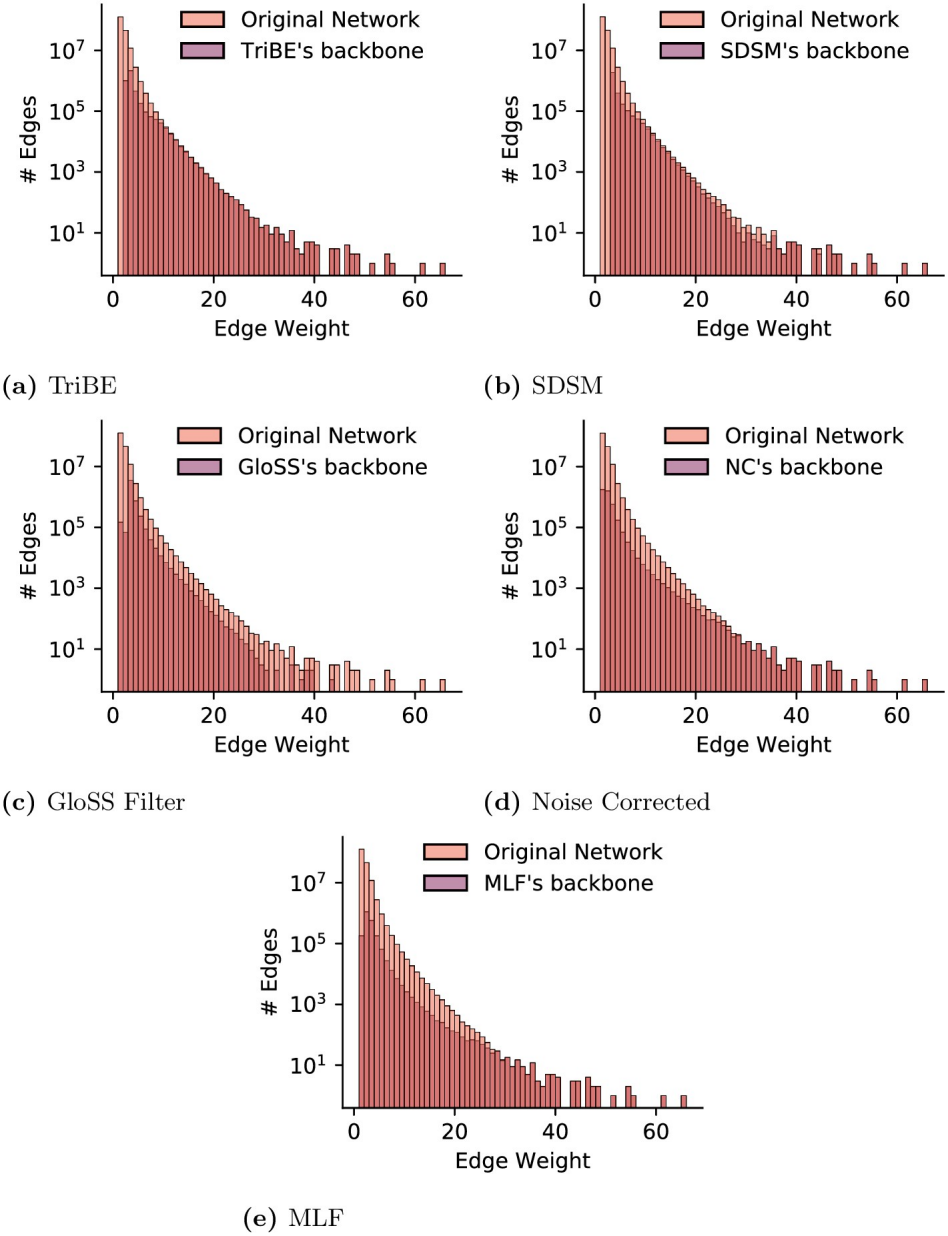
Online discussions on Instagram: Weight distribution for edges retained in the backbone by each method (distribution for original/complete network shown for comparison).

Since the methods extract backbones with different topological structures, we further analyze how each method deals with edges with respect to their weights. Fig 4 shows the distributions of weights for edges retained in the backbone by each method. Each plot also shows the distribution of edge weight in the original network for comparison. TriBE and SDSM (Fig 4a and 4b) remove many edges with small weights as well as some edges with large weights. GloSS Filter (Fig 4c) is more aggressive towards heavy edges and removes some of the edges over the whole range of values. Note that this method filters out all heaviest edges. NC (Fig 4d) is the most conservative method with respect to heavy edges and removes a smaller fraction of the edges through the whole range of weight values. MLF (Fig 4e) follows a similar pattern as NC, preserving heaviest edges. In summary, the results show that while all five methods share similarities (e.g., they all capture the effects of user activity levels and post popularity), they have their peculiarities when it comes to identifying an edge as salient, and they produce quite different backbones.

### 5.5 Step 4—Backbone evaluation

#### 5.5.1 Topological evaluation

We delve further into results of Table 2, comparing the backbones using the topological metrics. We focus on the modularity, since it gives us information about the communities, i.e., graphs representing collective patterns. GloSS Filter, for instance, produces the smallest improvement with respect to the original network (the modularity increases from 0.25 to 0.32), while NC and MLF show the largest improvements (from 0.25 to 0.61 and 0.62, respectively).

#### 5.5.2 Contextual evaluation

We shift to the quality of the backbones from a contextual point of view. We build regression models considering the following key assumption: *If two users i_1_ and i_2_ engage in the same online discussions, then the individual activities (comments) performed by each user are strongly correlated with the joint activities performed by both users.* If, however, comments posted by one user (or both) are mostly reactions to popular content or to some automatic tool (e.g., advertising or personalization mechanisms), or simply sporadic behavior, the activities of the user individually are only weakly correlated with the joint behavior of both users.

Based on this assumption, we build a regression model for each backbone and the original network. Given the edge weight *γ*(*i*_1_, *i*_2_) as dependent variable, the explanatory covariates are: (i) number of posts that user *i*_1_ commented on, (ii) number of posts that user *i*_2_ commented on, (iii) number of influencers that user *i*_1_ commented on, and (iv) number of influencers that user *i*_2_ commented on. We capture user activities by considering both the number of influencers and the number of posts each user commented on because it is often the case that the same influencer has multiple posts on different topics, each one attracting a different group of users (community) [22, 23]. Thus, we expect that only edges representing user interactions driven by joint engagement to be reasonably well explained by these covariates. Thus, the better the fitting of the model to the edge weights in a backbone, the better the quality of this backbone from a contextual perspective.

We check whether the covariates are linearly related to the dependent variable, a key assumption to use a linear regression model. We found that such linear relationship exists if a log transformation is applied to all covariates and to the dependent variable. Such transformation is often employed in variables with very skewed distributions, which is the case of edge weights (see Fig 4) and measures of user activity in social media applications [114, 115].

Table 3 shows the results of the model fitting for the five backbones and the original network. We assess the quality of model fitting using the coefficient of determination *R*^2^ and the NMRSE for the training edges and the NMRSE for the test edges.

**Table 3:** Online discussions on Instagram: Contextual evaluation of backbones by regression analysis.

Network Model	Training edges		Test edges (20% of common edges)
	R^2^	NRMSE	NRMSE
Original network	0.25	0.48	0.69
TriBE’s backbone	0.82	0.18	0.20
SDSM’s backbone	0.91	0.13	0.15
GloSS’s backbone	0.34	0.26	0.39
NC’s backbone	0.52	0.44	0.58
MLF’s backbone	0.49	0.35	0.51

Focusing first on the results for the training edges, we see that both SDSM and TriBE achieve significant improvements over the original network, both in terms of *R*^2^ and NMRSE. Indeed, these two methods are able to filter out many noisy edges and retain those more closely related to the phenomenon under study, which is reflected in the covariates used to build the regression models. SDSM and TriBE, by explicitly taking into account both the users’ activity level and the posts’ popularity when building the reference model, lead to high *R*^2^ values of 0.91 and 0.87, and an NRMSE of only 0.13 and 0.18. In turn, the other three methods, NC, MLF and GloSS, despite filtering out many edges (as shown in Table 2), lead to only moderate improvements over the original network.

The same conclusion holds for the test edges. Compared to the original network, the fits for both SDSM and TriBE show a notable reduction in NRMSE. This suggests that the edges considered salient by NC, MLF, and GloSS deviate the most from the common set of edges considered salient by all methods. This observation, in turn, suggests that these three methods retain a large fraction of possibly non-salient edges, which ultimately affects the fitting of the regression model.

SDSM performs slightly better than TriBE. Since we are only studying 8 popular influencers talking about politics, the effect of the third part (influencers) explicitly captured by TriBE but ignored by SDSM is mostly marginal. We expect a greater difference between the two methods, favoring TriBE for scenarios with a larger diversity of influencers in terms of both popularity and topics of posts.

In conclusion, our results indicate that evaluating backbone quality based solely on a single perspective may be misleading. For example, Table 2 shows that the backbones extracted from NC and MLF have the highest modularity scores. Yet, the regression analysis shows that the edges identified as salient by both, although well structured into strongly connected communities, do not offer a clearer understanding of the user behavior patterns driving the online discussions than the (poorly structured) original network. SDSM and TriBE, in turn, stand out as the best approach when considering a tradeoff between the quality of their communities and the ability of the selected edges to capture the user interactions that are driving the online discussions.

## 6 Case study 2: Coordinated behavior on WhatsApp

### 6.1 Characterization of the phenomenon

Our second case study concerns coordinated actions to disseminate information in WhatsApp groups. The platform connects users in end-to-end as well as group conversations. Despite being limited to only 256 simultaneous members, WhatsApp groups have been shown to be effective channels for the large dissemination of information [21, 76, 116, 117], notably misinformation [21, 118]. We here adopt the following widely used definition of *coordination* of users [20, 119, 120]: coordinated users typically exhibit a repetitive and synchronized pattern of activity.

Our present investigation relies on a dataset of anonymized messages shared in publicly accessible political-oriented WhatsApp groups in Brazil [21, 76], originally collected by the WhatsApp Monitor [116]. We focus our analysis on the month of the general presidential election in Brazil (October 2018), a time of great political mobilization and strong evidence of message coordination and orchestration in WhatsApp [21, 76, 116, 121, 122]. In summary, we analyze 4341 users who participated in 155 groups and shared 91417 unique pieces of information, in the form of text messages, images, audios and videos.

### 6.2 Step 1—Building the network model

We use the same network model adopted in [21, 76], referred to as *media-centric* network, which is defined as an undirected and weighted graph *G*_*WhatsApp*_ = (*V*, *E*) such that:

- *V* is the set of nodes representing users who shared at least one message in one of the monitored groups during the period of analysis;
- *E* represents the set of edges, where each edge connects two users if they share similar content in the same or different groups. The similarity between message content was estimated using a set of heuristics for filtering and identifying (near-)duplicate content. We refer the reader to [21] for more details on these heuristics. Each edge is weighted by the number of times the two users shared similar content.

In light of the adopted definition of coordination, salient edges are those whose weights are unusually high. The network may contain several noisy edges due to sporadic or weak interactions. For example, endogenous factors (e.g., temporary common interest or even large popularity of some particular content) may cause different users to share similar content, which may overshadow the actions of coordinated users who regularly and repeatedly share the same content. Therefore, the interest is to separate users who persistently engage in such common sharing from users who only sporadically exhibit such behavior. This separation implies favoring as salient those edges with heavier weights, either taking a local perspective (e.g., other edges incident to the same two nodes) or a global perspective (i.e., all edges in the network). This principle is used as a guideline in selecting candidate backbone extraction methods, as discussed next.

### 6.3 Step 2—Selection of candidate backbone extraction methods

We aim at selecting methods that explore the heterogeneity of the network by identifying as salient the edges with unusually heavier weights, based on individual (local) or network (global) patterns, as representative of persistent and repetitive interactions. As argued in Section 4.2.1, Threshold, HSS and RECAST are global methods that explore the heterogeneity of the edge weight distribution, favoring as salient the edges with heavier weights in the whole network. From a local perspective, Polya Urn Filter and Disparity Filter (DF) select as salient those edges whose weights are heavier than the weights associated with a subset of the edges (e.g., edges incident to the same pair of nodes). Thus, we define the set *C* of candidate methods as *C* = {Threshold, HSS, RECAST, Poly Urn and DF}. Recall that both Threshold and HSS explore structural properties, whereas the other three methods rely on statistical reference models to identify the salient edges.

### 6.4 Step 3—Backbone extraction

Table 4 summarizes the topological characteristics of the original network and the backbones extracted by each candidate method. The candidate methods have different parameters. Disparity Filter (DF), Polya Urn Filter (Polya) and RECAST require a *a*, which we set to 0.05, as shown in Table 7 in Appendix B. The Polya Urn method also requires a second parameter *a* related to the heterogeneity of the network. This parameter was set to 0.25, following a fine-tuning process, as briefly mentioned in Appendix A. The High Salient Skeleton (HSS) and Threshold approaches, on the other hand, take an arbitrary threshold value *τ* as input parameter. In both cases, we select *τ* to retain the top-*k*% most salient edges. Table 4 shows results for values of *τ* corresponding to the top 5% edges but we also tested for other values of *k* (thus of *τ*), as reported in Table 7. According to Table 4, the fractions of edges retained by both HSS and Threshold are slightly below 5%. This discrepancy is due to the removal of very small components (up to 3 nodes) of the backbone and the original network.

**Table 4:** Coordinated behavior on WhatsApp: Topological metrics of the network and backbones extracted by each candidate method (Columns 2-3 contain total numbers for the original network and also corresponding percentages for backbones).

Network Model	Nodes	Edges	Avg. Deg.	Density	Avg. Clust.	# C.C.	# Comm.	Mod.
Original network	4341	221002	103	0.0241	0.62	4	15	0.25
DF’s backbone	800 (18.7%)	9962 (4.51%)	24	0.0312	0.59	4	13	0.48
Polya’s backbone	734 (17.1%)	10527 (4.76%)	28	0.0391	0.59	5	15	0.48
Threshold’s backbone	495 (11.5%)	9489 (4.29%)	38	0.0776	0.73	3	8	0.45
RECAST’s backbone	313 (7.3%)	1875 (0.85%)	11	0.0384	0.49	2	7	0.37
HSS’s backbone	4281 (100%)	10996 (4.98%)	5	0.0012	0.14	4	29	0.44

In each graph, nodes belonging to the same community are represented by the same color, and edge weights are represented by both edge thickness and color (heavier/lighter edges are colored in red/blue).

As shown in the table and the graphs, DF, Polya and Threshold retain somewhat similar fractions of nodes (11-18%) and edges (4%) in the extracted backbones. Despite such large removal of nodes and edges, all three backbones have a number of connected components that approximate the original network. Interestingly, we also find that these backbones have larger density (especially the backbone extracted by Threshold) and comparable (if not higher) average clustering coefficient to the original network. RECAST, in turn, is the most aggressive method, retaining only around 7% of the original nodes and fewer than 1% of the original edges. This leads to a smaller number of components and average clustering coefficient, though the backbone’s density is still comparable to that of the DF’s and Polya’s backbones. At the other extreme, HSS extracts a rather large backbone while preserving all the original nodes. This backbone has a very different topological structure than the others, being much sparser and exhibiting lower density, degree, and average clustering.

Turning to the analysis of the communities (3 rightmost columns of Table 4), we observe that the number of communities varies considerably, being smaller for the backbones extracted by Threshold and RECAST. The former may be a consequence of the much denser backbone, while the latter may be related to the smaller number of nodes in the backbone. Recall that we still need contextual information to investigate the extent to which such communities are representative of the phenomenon.

**Fig 5:**
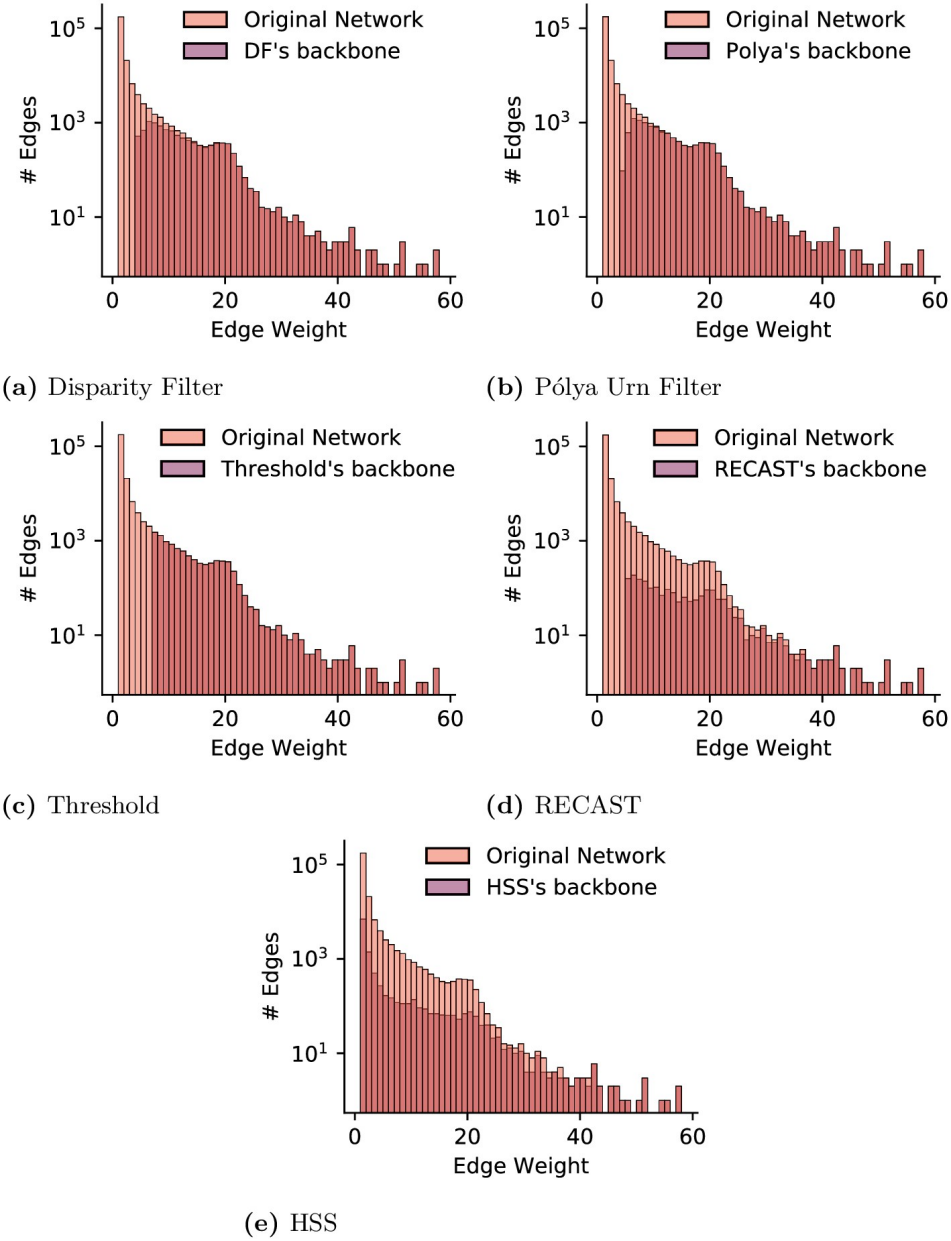
Coordinated behavior on WhatsApp: Weight distribution for edges retained in the backbone by each method (distribution for original/complete network shown for comparison purposes).

To further understand how the selected methods work, we analyze the distribution of edge weights, reported in Fig 5. All methods except HSS remove mostly edges with small weights. In particular, all edges with weights between 1 and 3 are removed by all four methods. HSS, in contrast, removes large fractions of edges across the whole range of weight values. As we will see in the next section, the HSS backbone is inferior to the others from a contextual perspective and, therefore, in terms of how well it captures edges related to the phenomenon under study.

### 6.5 Step 4—Backbone evaluation

#### 6.5.1 Topological evaluation

As in our first case study, the results presented in Table 4 show that all backbones are composed of more strongly connected and more clearly discriminated communities than the original network. The improvements in community structure, as captured by the modularity metric, are particularly large for the DF, Polya Urn and Threshold approaches. The backbones extracted by these methods are mostly composed of well structured communities of users who repeatedly share the same content, which favors the information spread at large. RECAST and HSS, in turn, produce backbones with weaker community structures.

#### 6.5.2 Contextual evaluation

The following key assumption guides our contextual evaluation of the backbone extraction methods: *If two users i_1_ and i_2_ are acting in coordination to share the same pieces of content repeatedly, such coordination should be reflected in their sharing patterns and user activity.* Guided by this assumption, we build a regression model where the dependent variable *γ*(*i*_1_, *i*_2_) is related with the following 11 explanatory variables: (i) total number of messages shared by *i*_1_ (*i*_2_); (ii) number of distinct messages shared by *i*_1_ (*i*_2_); (iii) number of messages with new content introduced (i.e., shared first) by *i*_1_ (*i*_2_); (iv) number of groups *i*_1_ (*i*_2_) participates in (inferred by the groups he/she shared content at least once); (v) Gini index of the number of messages shared by *i*_1_ (*i*_2_) across different groups; and (vi) number of common groups both *i*_1_ and *i*_2_ participate in. All variables, but the last one, are computed separately for *i*_1_ and *i*_2_, thus contributing as two covariates to the model. These variables capture different facets of user activity. The only variable related to the joint behavior of both users is the number of common groups, which indirectly captures whether or not the two users act in the same subset of observed groups. For example, users may act in a coordinated manner by frequently sharing the same content (i.e., heavy edge), even though the number of groups in which both participate is small. This could indicate, for example, that each of them forwards the same content to a particular subset of groups. As in our first case study, we also tested for the assumption of linearity, finding that it holds reasonably well after a square root transformation is applied to all covariates and the dependent variable.

Table 5 summarizes the results of the model fitting for each backbone and the original network for both training and test edges. Compared to our first case study, the fittings are generally poorer (note the lower *R*^2^ values in the training edges). We emphasize that it is much more challenging to perform a contextual evaluation of the network and backbone structures in this case study because we are focusing on messages shared in only 155 groups. All monitored groups belong to the same context (political domain) and are strongly interconnected as many users belong to multiple groups. However, these groups offer only a partial view of WhatsApp. The same users might participate in other groups, where they share and forward content, contributing to the information spread at large (see [116]). Thus, our analysis is limited by the lack of an unknown number of edges that most probably exist in the real underlying network connecting these users.

**Table 5:** Coordinated behavior on WhatsApp: Contextual evaluation of backbones by regression analysis.

Network Model	Training edges		Test edges (20% common edges)
	R^2^	NMRSE	NMRSE
Original network	0.21	1.33	0.92
DF’s backbone	0.35	0.40	0.53
Polya Urn’s backbone	0.30	0.51	0.55
Threshold’s backbone	0.22	0.37	0.51
RECAST’s backbone	0.22	0.51	0.51
HSS’s backbone	0.44	1.25	0.71

Under this constraint, the contextual evaluation leads to results consistent with those of topological evaluation. HSS has the highest *R*^2^, but also the highest errors (NMRSE), which are almost similar to the original network. The backbones extracted by DF, Polya Urn and Threshold, have the lowest errors in both the training and test sets. Concerning *R*^2^ the results are generally poor, with DF and Polya Urn performing better. Threshold performs worse than DF and Polya, suggesting that a global approach may leave out some important edges for the investigation. Indeed, as shown in Fig 5, both DF and Polya retain a more diversified set of edges in terms of edge weights.

In sum, we find that both DF and Polya Urn are the best methods to uncover evidence of user coordination when sharing similar content on WhatsApp. If one has to choose a method, DF is possibly the best choice as it contextually reveals communities closer to the phenomenon.

## 7 Conclusions and future work

We have proposed a principled methodology to select and evaluate the methods for extracting the network backbones that accurately represent collective behavior patterns in social media applications. In a fundamental and systematic way, this effort contributes to several recent network-oriented studies relying on backbone extraction strategies. In particular, this work fills a gap in the literature by emphasizing the need to: (1) carefully match assumptions and properties of the method with characteristics of the given phenomenon, showing that different methods may indeed extract quite different backbones, some of which offering little (if any) useful knowledge to the study; and (2) consider different criteria to evaluate the quality of alternative backbones, particularly in the absence of ground truth.

We offered a reasoned characterization of ten state-of-the-art backbone extraction methods, discussing their assumptions, properties and issues one must consider to apply them in practice. Such characterization advances existing knowledge available in the literature and is meant to help one in the selection of candidate methods for a target study. We propose alternatives to validate the extracted backbones, both structurally (based on topological measures extracted from the network) and contextually (based on phenomenon-specific attributes).

We applied our methodology to two very different scenarios that require different solutions and illustrate the complexity of selecting adequate methods for backbone extraction. In both scenarios, we found that some methods extract quite poor backbones. In contrast, others are suited to capture and describe the phenomenon under study, taking into account a trade-off between topological and contextual measures. We hope this work contributes by highlighting and demonstrating the risks of applying unfit and inadequate backbone extraction techniques.

A natural extension of this work is the application of the proposed methodology to other case studies. In particular, we hope that our methodology can foster the systematic evaluation of novel backbone extraction methods so as to broaden the range of alternative solutions, especially to study phenomena in social media applications, which have recently gained major importance to society. Going beyond the social media realm, our methodology can be applied to other domains. Examples include: (i) cultural mapping of communities by analyzing people’s visits to different places driven by the need to pursue cultural interests; (ii) coordinated actions in co-authorship or citation networks; (iii) collective economic changes in stocks or cryptocurrency market as a result of successive changes in different financial assets. An orthogonal direction to be pursued is the extension of the methodology to explore the temporal dimension, notably investigating strategies to accurately capture the temporal dynamics of the backbones and their communities.

## 8 Appendices

### A Backbone Extraction Methods Summary

**Threshold-based backbone extraction**. one of the simplest, most intuitive and most used methods [69–71, 74]. It consists on removing edges whose weights are smaller (or higher) than a pre-defined threshold *τ*, that is, edge saliency refers simply to edge weight. This method is adequate to studies where the salient edges are those with higher (or lower) weights. Otherwise, as previously argued [72], thresholds may bias the analysis and lead to misinterpretation of the results.

**High Salient Skeleton (HSS)** [32]. the backbone is extracted by first normalizing the edge weights and then computing shortest-path trees from each node to all other nodes in the network. Edge saliency is defined based on the frequency of its occurrence in the shortest path trees: edges with frequency below a pre-defined threshold *τ* are disregarded. In doing so, this method attempts to capture edges that simultaneously have heavy weights and are fundamental for keeping nodes connected. As such, the notion of edge saliency is inherently connected to network topology. Moreover, like for the threshold-based method, the use of a global threshold may lead to biases and misinterpretation [32].

**Disparity Filter (DF)** [77]. it assumes that an edge connecting a given pair of nodes is salient if it has a disproportionate weight compared to the other edges leading from the nodes to their respective neighbors. In other words, salient edges are those whose weights deviate significantly from the null hypothesis that the weights of all edges incident to a given node are uniformly distributed.

**Polya Urn Filter** [36]. similarly to DF, this method assumes that edge weights emerge from the aggregate process of individual nodes’ preferences to interact with each other over time. It also assumes that interactions between nodes are maintained and reinforced, such that the larger the number of interactions between the same two nodes, the higher the probability of they interacting again. A reference model is built for each edge, using the Polya Urn model [123] which captures the reinforcement of existing interactions by examining the degree and strength (the sum of the weights of all edges incident to the node) of each node incident to this edge. This reinforcement mechanism can be regulated and estimated by the system through a fine-tuning process. Salient edges are those that deviate significantly from such reference model (according to a given *alpha*).

**Marginal Likelihood Filter** (MLF) [35]. assumes that edge saliency should be analyzed in light of the strengths of the two nodes the edge connects. The higher the strengths the larger the edge weight must be to be considered salient. Specifically, the method builds a reference edge weight distribution model for each edge: the probability that edge between nodes *i* and *j* ends up with weight *w*_*ij*_ is based on a Binomial distribution with parameters *n* defined by the total strengths of all nodes in the network and *p* computed based on the strengths of nodes *i* and *j*. An edge is considered salient if the observed weight deviates significantly from the one predicted by the reference model.

**Noise Corrected (NC)** [33]. similarly to DF and the Polya Urn Filter methods, NC also assumes that edge saliency arises from the cooperation between nodes. However, unlike those methods, NC preserves peripheral-peripheral connections, which is crucial for capturing edges that, despite having small weights, may still be considered relevant for the phenomenon under study. These connections may be preserved by estimating the expectation and variance of edge weights using a hypergeometric distribution, taking into account the propensity of both nodes to send and receive edges. It also provides a direct approximation through a per-edge reference Binomial distribution (similarly to the MLF method). The main advantage of NC, though, is the ability to estimate an error for the expectation of the weights. As in the other methods, an edge is considered salient if its observed weight significantly exceeds the expected weight (given the strengths of both nodes).

**Global Statistical Significance (GloSS) Filter** [31]. it assumes that salient edges cannot be identified independently of the overall network topology, once nodes have different degrees. As such, it builds a single (null) reference model that preserves the edges between nodes as well as the overall edge weight distribution. Yet, when selecting salient edges, i.e., edges whose observed weights significantly deviate from the reference model, the method estimates the probability of observing an edge weight between two given nodes considering the nodes’ observed degrees and strengths as constraints.

**Tripartite Backbone Extraction (TriBE)** [22, 23]. this method was proposed to study phenomena driven by user interactions in social media applications. It exploits the tripartite structure commonly found in such platforms, that is, a piece of content, the content creator, and the other users (e.g., the followers) who interact with each other in reaction to that content (e.g., by commenting on a post, retweeting the same tweet, etc). As such, the method addresses the heterogeneity in user activity level and content popularity typically observed in social media applications. Specifically, it builds a reference weight distribution model for each edge, based on a Poisson binomial distribution, whose parameters are computed based on the distributions of content popularity and user engagement towards content from the same creator (as estimated by prior interactions). Once again, salient edges are those whose observed weights significantly deviate from their corresponding reference models.

**Stochastic Degree Sequence Model (SDSM)** [37]. This method considers a bipartite network to evaluate the salience of an edge in the projection. The SDSM specifies the degree sequence of agents and artifacts of a bipartite network on the average constraint such that their expected row sums and expected column sums are equal to the observed ones. In this way, a reference model is built based on the Poisson binomial distribution that selects as salient those edges whose observed weights differ significantly from the expected value given the degree sequence constraints.

### B Parameter sensitivity analysis

We report in this appendix additional results from a sensitivity analysis we performed to parameterize the backbone extraction methods. Table 6 shows results of the impact of the *alpha* (input parameter) on the methods selected as candidates for the study of online discussions on Instagram (case study 1 reported in Section 5). Table 7 shows corresponding results for the methods selected as candidates for the study of coordinated behavior on WhatsApp (case study 2, discussed in Section 6).

**Table 6:** Online discussions on Instagram: Impact of method parameters on topological and contextual metrics.

Method	% N	% E	# Comm.	Mod.	Parameter		*R* ^2^	NMRSE
TriBE	99.86	9.43	6	0.43	*alpha*	0.1	0.80	0.18
TriBE	99.31	2.19	11	0.56		0.05	0.82	0.18
TriBE	92.97	0.48	18	0.73		0.01	0.85	0.18
TriBE	62.43	0.14	93	0.81		5e-3	0.91	0.21
TriBE	28.32	0.03	44	0.74		1e-3	0.95	0.18
TriBE	21.64	0.02	38	0.77		5e-4	0.96	0.17
TriBE	12.40	0.01	45	0.86		1e-4	0.96	0.19
SDSM	99.87	25.07	7	0.27	*alpha*	0.1	0.60	0.23
SDSM	99.54	15.71	8	0.30		0.05	0.80	0.18
SDSM	94.22	5.43	8	0.38		0.01	0.91	0.14
SDSM	92.66	4.61	9	0.40		0.005	0.93	0.13
SDSM	77.40	1.47	12	0.46		0.001	0.91	0.13
GloSS	68.29	2.58	7	0.32	*alpha*	0.1	0.34	0.26
GloSS	65.45	0.73	6	0.39		0.05	0.65	0.28
GloSS	58.59	0.27	7	0.58		0.01	0.71	0.32
GloSS	56.44	0.18	7	0.70		0.005	0.81	0.37
NC	100.00	63.74	5	0.39	*alpha*	0.1	0.28	0.46
NC	100.00	47.20	5	0.49		0.05	0.27	0.48
NC	100.00	23.93	5	0.59		0.01	0.21	0.52
NC	100.00	18.96	6	0.57		0.005	0.21	0.54
NC	98.10	11.52	6	0.52		0.001	0.28	0.52
NC	95.80	9.12	8	0.52		5e-4	0.33	0.51
NC	86.20	5.17	9	0.56		1e-4	0.41	0.48
NC	81.18	4.06	10	0.57		5e-5	0.45	0.47
NC	69.24	2.26	8	0.61		1e-5	0.52	0.45
NC	64.88	1.73	10	0.62		5e-6	0.53	0.44
NC	57.31	0.96	8	0.67		1e-6	0.55	0.42
NC	54.96	0.76	8	0.68		5e-7	0.56	0.42
NC	50.16	0.44	9	0.69		1e-7	0.60	0.41
NC	47.95	0.35	11	0.69		5e-8	0.62	0.41
NC	41.99	0.21	16	0.70		1e-8	0.65	0.40
NC	39.21	0.17	19	0.70		5e-9	0.67	0.40
NC	32.96	0.11	22	0.70		1e-9	0.70	0.40
NC	30.54	0.09	20	0.69		5e-10	0.71	0.40
NC	25.63	0.06	22	0.69		1e-10	0.74	0.39
MLF	98.79	16.16	7	0.51	*alpha*	0.1	0.17	0.54
MLF	94.78	10.27	19	0.49		0.05	0.28	0.49
MLF	74.61	3.95	8	0.55		0.01	0.47	0.38
MLF	65.75	2.71	9	0.54		5e-3	0.50	0.36
MLF	49.78	1.16	12	0.62		1e-3	0.49	0.35
MLF	46.82	0.85	10	0.63		5e-4	0.52	0.35
MLF	43.95	0.45	12	0.63		1e-4	0.63	0.35

**Table 7:** Coordinated behavior on WhatsApp: Impact of method parameters on topological and contextual metrics.

Method	% N	% E	# Comm.	Mod.	Parameter		*R* ^2^	NMRSE
DF	4.30	0.33	10	0.55	*alpha*	0.001	0.33	1.31
DF	8.34	0.91	13	0.54		0.005	0.30	1.30
DF	10.77	1.51	13	0.52		0.010	0.31	1.31
DF	18.69	4.51	13	0.48		0.050	0.35	1.47
DF	26.26	7.23	13	0.45		0.100	0.34	1.63
Polya Urn	7.62	1.21	10	0.50	*alpha*	0.001	0.23	1.11
Polya Urn	10.35	2.10	11	0.50		0.005	0.27	1.20
Polya Urn	11.82	2.66	12	0.49		0.010	0.29	1.26
Polya Urn	17.15	4.76	15	0.48		0.050	0.30	1.43
Polya Urn	19.36	6.20	13	0.45		0.100	0.30	1.52
Threshold	3.22	0.45	6	0.43	Threshold/Percentile	99.5	0.38	0.87
Threshold	4.06	0.94	6	0.41		99.0	0.31	0.82
Threshold	11.56	4.29	8	0.45		95.0	0.22	1.36
Threshold	19.55	8.13	12	0.42		90.0	0.24	1.65
Threshold	26.91	11.17	12	0.37		80.0	0.24	1.81
RECAST	2.80	0.14	9	0.38	*alpha*	0.001	0.34	2.06
RECAST	2.80	0.14	8	0.38		0.005	0.34	2.06
RECAST	2.80	0.14	8	0.38		0.010	0.34	2.06
RECAST	7.31	0.85	7	0.37		0.050	0.22	1.97
RECAST	7.31	0.85	8	0.36		0.100	0.22	1.97
HSS	22.78	0.36	176	0.98	Threshold/Percentile	99.5	0.36	0.89
HSS	51.23	0.91	198	0.96		99.0	0.29	1.36
HSS	100.00	4.98	29	0.44		95.0	0.44	2.71
HSS	100.00	9.93	21	0.31		90.0	0.46	2.56
HSS	100.00	19.98	18	0.33		80.0	0.48	2.40

As pointed out in Section 4, defining such parametrization leads to a multicriteria problem. To guarantee the existence of a common set of edges under the notion of saliency consensus between the methods, we adopted the following strategy. In both scenarios, we based the most aggressive method in terms of the number of removed edges in the backbone and adjusted the parameters so that each method maintains an approximate percentage of edges in the backbone. For the first case study in Table 6, this bound was based on the GloSS Filter retaining 0.18% of the edges in the backbone. However, keeping this approximate percentage of edges for all methods does not allow us to create the minimum common set. To account for this, we parameterized the GloSS Filter with *alpha* = 0.1. Consequently, we look for parameters within the tested values for all methods that hold approximately 2.58% edges in the backbone. We found that such parameterization is detrimental to the GloSS Filter, but on the other hand, it allows us to create such a set that is representative of a consensus among methods with respect to a set of edges that are considered salient.

For the second case study, presented in Table 7, we started with RECAST, which is quite aggressive and consequently *alpha* = 0.05. Note that RECAST produces a global discrete distribution and thus some *alpha* values do not change the backbone extraction. Thus, we assumed that each method has about 0.85% of the edges in the backbone. However, with such a restrictive value, we could not create a common set. After incrementally increasing each method and assuming that this was the limit for RECAST, we found that a percentage of 4% to 5% of edges would satisfy this requirement.
